# Cytokine profiles by peripheral blood monocytes are associated with changes in behavioral symptoms following immune insults in a subset of ASD subjects: an inflammatory subtype?

**DOI:** 10.1186/s12974-014-0187-2

**Published:** 2014-10-27

**Authors:** Harumi Jyonouchi, Lee Geng, Amy L Davidow

**Affiliations:** Department of Pediatrics, Saint Peter’s University Hospital, 254 Easton Ave., New Brunswick, NJ 08873 USA; Departments of Public Health, New Jersey Medical School-Rutgers University, 185 South Orange Ave., Newark, NJ 07101 USA

**Keywords:** ASD, inflammatory subtype, NFA, GI symptoms, Cytokines, Neuroimmune network

## Abstract

**Background:**

Some children with autism spectrum disorders (ASD) are characterized by fluctuating behavioral symptoms following immune insults, persistent gastrointestinal (GI) symptoms, and a lack of response to the first-line intervention measures. These children have been categorized as the ASD-inflammatory subtype (ASD-IS) for this study. We reported a high prevalence of non-IgE mediated food allergy (NFA) in young ASD children before, but not all ASD/NFA children reveal such clinical features of ASD-IS. This study addressed whether behavioral changes of ASD-IS are associated with innate immune abnormalities manifested in isolated peripheral blood (PB) monocytes (Mo), major innate immune cells in the PB.

**Methods:**

This study includes three groups of ASD subjects (ASD-IS subjects (N = 24), ASD controls with a history of NFA (ASD/NFA (N = 20), and ASD/non-NFA controls (N = 20)) and three groups of non-ASD controls (non-ASD/NFA subjects (N = 16), those diagnosed with pediatric acute onset-neuropsychiatric syndrome (PANS, N = 18), and normal controls without NFA or PANS (N = 16)). Functions of purified PB Mo were assessed by measuring the production of inflammatory and counter-regulatory cytokines with or without stimuli of innate immunity (lipopolysaccharide (LPS), zymosan, CL097, and candida heat extracts as a source of β-lactam). In ASD-IS and PANS subjects, these assays were done in the state of behavioral exacerbation (‘flare’) and in the stable (‘non-flare’) condition. ASD-IS children in the ‘flare’ state revealed worsening irritability, lethargy and hyperactivity.

**Results:**

‘Flare’ ASD-IS PB Mo produced higher amounts of inflammatory cytokines (IL-1β and IL-6) without stimuli than ‘non-flare’ ASD-IS cells. With zymosan, ‘flare’ ASD-IS cells produced more IL-1β than most control cells, despite spontaneous production of large amounts of IL-1ß. Moreover, ‘flare’ ASD-IS Mo produced less IL-10, a counterregulatory cytokine, in response to stimuli than ‘non-flare’ cells or other control cells. These changes were not observed in PANS cells.

**Conclusions:**

We observed an imbalance in the production of inflammatory (IL-1ß and IL-6) and counterregulatory (IL-10) cytokines by ‘flare’ ASD-IS monocytes, which may indicate an association between intrinsic abnormalities of PB Mo and changes in behavioral symptoms in the ASD-IS subjects.

## Background

ASD are a behaviorally defined syndrome that consists of a heterogeneous group of neurodevelopmental disorders [1]. Various gene mutations or microdeletion syndromes are known to cause ASD behavioral symptoms; however, single gene mutations are likely to account for only a small fraction of ASD cases [2]. In the majority of ASD subjects, no causative gene mutations have been identified. In these cases, it is more likely that complex interactions among multiple genetic and environmental factors contribute to the onset and progress of ASD, as seen in other polygenic diseases.

Currently, first-line treatment measures for ASD are behavioral and pharmacological interventions. Unfortunately, favorable responses to these measures are not universally achieved; some ASD patients respond well to these measures, while others do not. This is partly attributed to both the heterogeneity of the ASD population [1] and a high frequency of comorbid medical conditions that may affect behavioral symptoms through pain and discomfort [3]. At this time, there are no objective biomarkers for identifying the children who will not be responsive to the first-line therapeutic measures.

Although there are alternative treatments, including those proposed by practitioners of complementary and alternative medicines (CAM) [4,5], these are not universally effective either, and research has not shown which are beneficial to what types (subgroups) of ASD subjects. There is also a lack of both reliable objective biomarkers and understanding of the mechanisms of actions [6]. Parents of ASD children may turn to such CAM treatments, hoping for the best. There is an urgent need for identifying objective biomarkers that may guide optimal effective measures for ASD children who fail to respond to the first-line intervention measures.

However, the etiology of poor responses is also multifactorial, and it is extremely difficult to identify biomarkers by focusing on ‘poor responders’ to conventional first line treatments. Previous genetic workups indicate that mutational analysis may not be practical for segregating ASD subjects who have an apparent involvement of multiple genes [1]. Instead, it may be more feasible to focus on ASD children who are not only poor responders but also have objective clinical and laboratory characteristics that are distinguishable from other ASD subjects. The ASD-IS children described in our previous studies, as detailed in the next paragraph, may be ideal for such a purpose

In our pediatric allergy/immunology (AI) clinic, we have evaluated a substantial number of ASD children for possible food allergy (FA) and underlying immune condition. Evaluations of these ASD children led us to recognize that some of them manifest clinical features indicating immunodysregulation and/or chronic inflammation. Namely, these children exhibit chronic and/or recurrent sino-pulmonary infection; adverse reactions to multiple medications; persistent GI symptoms with high frequency of NFA; and prolonged courses of illness. Moreover, these children do not respond well to pharmacological and behavioral intervention measures per parental report [7,8]. In this study, we categorized such ASD subjects as ASD-IS. Further studies of these children, using peripheral blood mononuclear cells (PBMCs), revealed abnormalities of innate immune responses that were distinguishable from both ASD and normal controls [9]. Changes in transcriptional profiles of isolated PB Mo, which did not overlap with those found in either the ASD and normal controls, were also found [9]. Based on these findings, we hypothesized that ASD-IS children have distinguishable innate immune abnormalities, which can be associated with behavioral changes following immune insults, typically microbial infection.

It should also be noted that our previous results revealed discrepancies between transcriptional profiles of PB Mo and protein levels of cytokine produced by PBMCs, a mixture of lymphocytes, PB Mo, and other lineage cells. Despite the upregulation of mRNA of various cytokines by PB Mo, the production of such cytokines by PBMCs was lower in ASD-IS subjects; stimulants used were targeted to PB Mo [9]. This could be associated with the influence of cells other than PB Mo, such as regulatory T cells (Treg cells) or suppressive (tolerizing) dendritic cells (DCs) contained in PBMCs. Alternatively, post-transcriptional regulation in PB Mo, such as those exerted by microRNA (miRNA) [10,11], may also affect the protein levels of cytokines depending on the stage of PB Mo activation. We hypothesized that if intrinsic post-translational regulation has a role in the discrepancy between protein and mRNA expression of cytokines, isolated PB Mo may reveal distinct patterns of cytokine production depending on the activated state of PB Mo. That is, post-transcriptional regulation exerted by miRNA and other factors are expected to change rapidly depending on the activated stage of PB Mo [10]. It should also be noted that such changes of post-transcriptional regulation may affect the neuroimmune network [12]. Interestingly, in our observation, ASD-IS children are characterized by rapid changes in behavioral symptoms following immune insults, as observed in PANS children [13].

To test our hypothesis, we examined cytokine production by purified PB Mo from ASD-IS and control ASD and non-ASD subjects. We have reported previously reported a high prevalence of NFA in young ASD children with chronic GI symptoms, including ASD-IS children [7]. Since the presence of NFA may indicate innate immune abnormalities that predispose to NFA at least in the gut mucosa [14], ASD and non-ASD controls were further separated into two subgroups, children with or without NFA. In addition, non-ASD/PANS subjects were added to the non-ASD control groups, since the ASD-IS children we recruited are often diagnosed with PANS by CAM practitioners prior to entrance into this study.

## Materials and methods

The study follows the protocols approved by the Institutional Review Board at Rutgers-New Jersey Medical School. Informed consent was obtained prior to blood sample obtainment.

### Study subjects

ASD subjects were recruited in the Pediatric Allergy/Immunology clinic. Diagnosis of ASD in the study subjects was made at various autism diagnostic centers, including ours. The ASD diagnosis was based on the Autism Diagnostic Observation Scale (ADOS) and/or the Autism Diagnostic Inventory-Revisited (ADI-R), and these diagnoses were verified by the medical records. For those whose ADOS and ADI-R records were not verified, the ADOS and/or ADI-R was administered to confirm the diagnosis.

Any subjects with impaired hearing/eyesight, any motor disability, such as cerebral palsy, or medical conditions with known gene mutations were excluded from the study. ASD subjects were also evaluated for their behavioral symptoms, sleep habits, and adaptive skills by using the Aberrant Behavior Checklist (ABC) [15], the Children’s Sleep Habits Questionnaires (CSHQ)[16], and the Vineland Adaptive Behavior Scale (VABS) [17], respectively. For ASD subjects with limited expressive language, the presence of physical pain was assessed by using the Non-communicating Children’s Pain Checklist (NCCPC) [18].

ASD subjects were further subdivided into three groups (ASD-IS, ASD/NFA, and ASD/non-NFA) and each group was defined as follows;

- *ASD-IS children*: ASD-IS children are defined as those with a history of fluctuating behavioral symptoms following immune insults (mainly microbial infection). Symptoms must have been documented by individuals other than parents, such as teachers/therapists, a minimum of three times. In addition, a history of persistent GI symptoms, often diagnosed as non-IgE mediated food allergy (NFA - see next section for diagnostic criteria), is required. Among the ASD-IS subjects, 14/24 subjects were diagnosed with food protein induced enterocolitis syndrome (FPIES), a severe form of NFA, prior to enrollment in this study, and two ASD-IS subjects were diagnosed with eosinophilic esophagitis (EoE) on the basis of biopsy results. These ASD-IS subjects reported to have had loss of once-acquired cognitive skills based on the reports of teachers, therapists and/or previous records of developmental assessment.
  - We defined ‘flares’ as worsening behavioral symptoms following immune insults, despite the resolution of acute conditions such as viral syndrome (that is, the resolution of other infectious symptoms if associated with a microbial infection, lack of fever, and no other acute physical symptoms associated with immune insults). Most of the immune insults in this study were clinically judged to be microbial infection (mainly viral syndrome). In ASD-IS children, we obtained samples at least once in the ‘flare’ and ‘non-flare’ states. Changes in behavioral symptoms by parental reports were confirmed by reports from teachers and other caregivers.
  - *ASD/NFA controls*: ASD children with a history of NFA were defined as ASD children with a clinical history of NFA that met the current diagnostic criteria of NFA as detailed in the section of non-ASD/NFA subjects. These subjects lacked the fluctuating behavioral symptoms observed in the ASD-IS children, following immune insults (mainly microbial infection).
- *ASD/non-NFA controls:* ASD subjects without a history of NFA or fluctuating behavioral symptoms following immune insults.

Non-ASD controls included 3 groups as indicated in the abstracts (Non-ASD/NFA subject, PANS subjects, and normal controls without NFA or PANS) and each group was defined as follows;

- *Non-ASD/NFA subjects*: Non-ASD subjects with a history of NFA were recruited in the pediatric A/I Clinic. NFA was diagnosed previously with the resolution of GI symptoms following implementation of a restricted diet (that is, avoidance of offending food) and recurrence of symptoms with the re-introduction of the offending food, following the Food Allergy Diagnostic Guidelines, as there is no specific histological finding in biopsy for NFA [19]. NFA patients are per definition, non-reactive to skin prick testing and negative for food allergen-specific serum IgE [19]. These non-ASD/NFA subjects did not have significant GI symptoms that would affect our assays at the time of sample obtainment. Most of the non-ASD/NFA subjects were diagnosed with food protein-induced enterocolitis syndrome (FPIES), a severe form of NFA, based on the clinical diagnostic criteria for NFA. Severe clinical symptoms of FPIES often manifested as failure to thrive in these subjects. FPIES, like other NFA, is known to have no specific histological findings [19]. One non-ASD/NFA subject was diagnosed with eosinophilic esophagitis (EoE) by biopsy [20], and this subject was negative for food allergen-specific IgE in the serum.
- *PANS subjects*: Children with PANS were diagnosed clinically on the basis of the current diagnostic criteria proposed by Swedo *et al*. [13]. PANS subjects were typically developing without previous history of developmental disability, learning disability, or any other neuropsychiatric conditions prior to onset of PANS symptoms.
- *Normal controls*: Typically developing, normal control children without ASD, PANS, or NFA were recruited in the pediatrics subspecialty clinic where our A/I clinic also operated. Attempts were made to match controls with ASD study subjects in terms of gender, age, and ethnicity.

### Diagnosis of asthma and allergic rhinitis

Allergic rhinitis (AR) and allergic conjunctivitis (AC) were diagnosed with positive skin prick test reactivity and/or presence of allergen-specific IgE in the serum accompanied by clinical features consistent with AR and AC [21,22]. Asthma diagnosis was based on the guidelines from the Expert Panel Report 3 [23]. Asthma without skin test reactivity to allergens and/or allergen-specific IgE antibodies was categorized as non-atopic asthma [22].

### Antibody deficiency syndrome

Specific polysaccharide antibody deficiency (SPAD) was diagnosed with detectable antibody (Ab) titers (more than 1.3 μg/ml) to fewer than 3 of 14 serotypes of *Streptococcus pneumonia* in response to Pneumovax™ [24], a standard diagnostic measure for SPAD.

Demographic information of the study subjects is summarized in Table 1 and comorbid conditions are summarized in Table 2.

**Table 1:** **Demographics of the study subjects**

	**Age (year)** ^**a**^	**Gender**	**Ethnicity**	**ASD** ^**b**^ **Diagnosis**		
	**Median (range)**	**(M:F)**		**Autism**	**ASD**	**PDD**
**ASD subjects**						
ASD-IS (N = 24)	11.8 (6.0-27.0)	19:5	1 Asian, 1 mixed, 22 W	17	6	1
ASD/NFA (N = 20)	7.5 (3.3-22)	15:5	2 Asians, 2 mixed, 16 W	11	4	5
ASD/non-NFA (N = 20)	12.9 (3.6-20.5)	17:3	1 AA, 3 mixed, 16 W	12	5	1^c^
**Non-ASD subjects**						
Non-ASD/NFA (N = 16)	4.3 (1.2-20.6)	9:7	16 W			
Normal control (N = 16)	10.5 (4.0-18.7)	10:6	2 mixed, 14 W			
PANS (N = 18)	8.2 (5.0-15.8)	18:2	18 W			

^a^Age when the subject enrolled in the study.

^b^Abbreviations used: AA, African-American; ASD, autism spectrum disorder; ASD-IS, ASD-inflammatory subtype; F, female; M, male; NFA, non-IgE mediated food allergy; PDD, pervasive developmental disorder, PANS, pediatric acute-onset neuropsychiatric disorder; W, Caucasian.

^c^In addition to one PDD subject, two ASD/non-NFA subjects were diagnosed with Asperger syndrome.

**Table 2:** **Comorbid conditions of the study subjects**

	**Seizure disorder**	**Autoimmune conditions**	**Allergic rhinitis**	**Asthma**		**Ab deficiency**
**ASD** ^**c**^ **subjects**				Atopic	Non-atopic	
ASD-IS (N = 24)	4/24	2/24^a^	2/24	0	3/24	3/24^b^
ASD/NFA (N = 20)	2/20	0	3/20	1/20	0	0
ASD/non-NFA (N = 20)	1/20	0	0	0	0	0
**Non-ASD subjects**						
Non-ASD/NFA (N = 16)	0	0	0	0	0	0
Normal control (N = 16)	0	0	3/16	1/16	1/16	0
PANS (N = 18)	0	0	3/18	0	0	2/18

^a^One subject was diagnosed with psoriasis and another subject was diagnosed with anti-phospholipid syndrome.

^b^All subjects diagnosed with antibody deficiency syndrome were on IVIG treatment at the time of sample obtainment. All the subjects were shown to have severely impaired responses to Pneumovax™.

^c^ *Abbreviations used*; Ab, antibody; ASD, autism spectrum disorder; ASD-IS, ASD-inflammatory subtype; NFA, non-IgE mediated food allergy; PANS, pediatric acute onset neuropsychiatric syndrome.

### Sample obtainment

PB samples were obtained by venipuncture after informed consent was obtained. Efforts were made to obtain the PB samples at the time of routine blood work in order to minimize the number of venipunctures. For most of the non-ASD/NFA, normal control, ASD/non-NFA, and ASD/NFA groups, only one blood sample was taken. For PANS and ASD-IS subjects, more than one blood sample was obtained in an effort to have blood samples taken during times when subject was in stable condition (‘non-flare’ samples) and when flare-ups of behavioral symptoms following immune insults (‘flare’ samples) occurred. Venipuncture was conducted by the physician, and if requested, the site of venipuncture was numbed by the application of topical lidocaine (Emla cream™).

### Cell cultures

PBMCs were isolated by Ficoll-Hypaque density gradient centrifugation. PB Mo were purified by negatively selecting PB Mo depleting T, B, natural killer, and DCs, using magnetic beads labeled with anti-CD3, CD7, CD16, CD19, CD56, CD123, and glycophorin A (Monocyte Isolation Kit II, human; MILTENYI BIOTEC, Cambridge, MA, USA).

Innate immune responses were assessed by incubating purified PB Mo (2.5×10^5^ cells/ml) overnight with toll-like receptor (TLR) 4 agonist (LPS; 0.1 μg/ml, GIBCO-BRL, Gaithersburg, MD, USA), TLR2/6 agonist (zymosan; 50 μg/ml, Sigma-Aldrich, St. Louis, MO, USA), TLR7/8 agonist (CL097, water-soluble derivative of imidazoquinoline, 20 μM, InvivoGen, San Diego, CA, USA), and dectin 1 agonist (heat killed *Candida albicans* as a source of ß-lactam (10^9^ cells/ml) - 10 μl/ml, InvivoGen) in RPMI 1640 with additives as previously described [25]. Overnight incubation was adequate to induce the optimal responses in this setting.

Levels of proinflammatory (tumor necrosis factor-α (TNF-α), interleukin-1ß (IL-1β), IL-6, IL-12p40, and IL-23) and counter-regulatory (IL-10, transforming growth factor-ß (TGF-ß) and soluble TNF receptor II (sTNFRII)) cytokines in the culture supernatant were then measured by enzyme-linked immunosorbent assay (ELISA). For ELISA, OptEIA™ Reagent Sets (BD Biosciences) were used for IFN-γ, IL-1ß, IL-5, IL-6, IL-10, IL-12p40, and TNF-α, and ELISA reagent set (R & D, Minneapolis, MN, USA) were used for sTNFRII, IL-17 (IL-17A), and TGF-ß. IL-23 ELISA kit was purchased from eBiosciences (San Diego, CA, USA). Intra- and intervariation of cytokine levels were less than 5%.

### Statistics

For comparison of test values with control values, a Wilcoxon rank sum test was used. Differences in frequency were tested with a Chi square (χ^2^) test and the correlation was tested using linear regression analysis. These tests were performed using R.3.1 (R-Development Core Team 2011). A *p* value of <0.05 was considered to be statistically significant.

## Results

### Clinical findings

The prevalence of comorbid conditions is summarized in Table 2. Seizure disorders requiring daily anti-seizure medications were reported in 4/24 ASD-IS, 2/20 ASD/NFA, and 1/20 ASD/non-NFA subjects, respectively (Table 2). None of the non-ASD subjects were diagnosed with seizure disorders. Autoimmune diseases were reported in only two ASD-IS female subjects (anti-phospholipid syndrome and psoriasis, respectively). Allergic rhinitis was reported in 2/24 ASD-IS, 3/20 ASD/NFA, 3/16 normal controls, and 3/17 PAN S subjects. Thus the prevalence of well-described autoimmune diseases or IgE mediated atopic disorders was not high in any of the study groups. In addition, the frequency of non-atopic asthma was not high in any of the study groups as well (Table 2). Antibody deficiency syndrome, as defined in the methods section, was diagnosed in 3/24 ASD-IS subjects and 2/18 PANS subjects.

**Figure 1:**
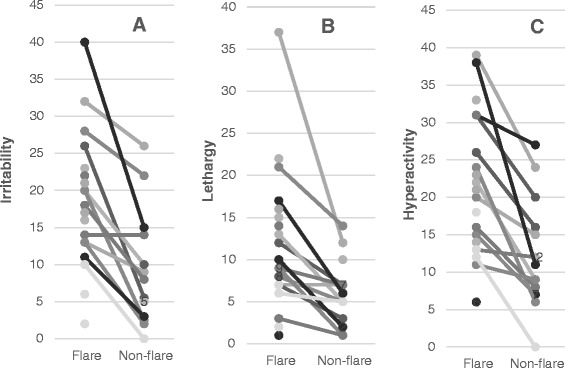
**In autism spectrum disorder-inflammatory subtype** **(ASD-IS) children, significant differences were observed in the scores of the Aberrant Behavior Checklist (ABC) subdomains I (irritability), II (lethargy), III (stereotypy), and IV (hyperactivity) between the ‘flare’ and ‘non-flare’ states (** ***P*** **<0.005 for subdomains I and IV and** ***P*** **<0.01 for subdomain II by a paired Wilcoxon rank sum test.**

A developmental evaluation using either the Woodcock-Johnson III test (WJIII) or school-reported IQ evaluation in the year prior to the enrollment revealed that the general intellectual ability (GIA) of most ASD subjects is <1st percentile rank. However, the GIA of a few ASD subjects ranked within the normal range (>5th percentile rank). One Asperger subject in the ASD/non-NFA group had a GIA ranked as the 98th percentile, although his adaptive skills were in the 6th percentile based on the VABS. There were no difference in scores of CSHQ, standardized adaptive skills composition by VABS, and subscale behavioral scores by ABC among the ASD subjects (Table 3). However, when ABC scores from the ASD-IS subjects were compared during the ‘flare’ versus ‘non-flare’ states as defined in the method section, ASD-IS subjects experiencing ‘flares’ revealed significantly higher scores in the ABC subdomains I (irritability) (*P* <0.005), II (lethargy) (*P* <0.01), and IV (hyperactivity) (*P* <0.005) (Figure 1).

**Table 3:** **Summary of the Aberrant Behavior Checklist (ABC), the Vineland Adaptive Behavioral Scale (VABS), and the Children’s Sleep Habit Questionnaire (CSHQ) results**

**ASD** ^**d**^ **study group**	**ABC subscale score** ^**a**^			**VABS PR <1** ^**c**^	**CSHQ**
	**I (irritability)**	**II (lethargy)**	**IV (hyperactivity)**	**Number (%)**	**Total score**
ASD-IS	13.7 ± 8.2^b^	8.9 ± 5.5	17.6 ± 8.2	18/24 (75%)	51.4 ± 5.0
ASD/NFA	15.1 ± 8.3	12.2 ± 9.8	21.1 ± 11.8	13/20 (65%)	49.7 ± 6.4
ASD/non-NFA	13.8 ± 9.6	10.1 ± 8.4	16.7 ± 10.1	16/20 (80%)	50.6 ± 7.2

^a^ABC subscales I, II, III in the ASD study groups were shown since ASD-IS children revealed changes in these scales during ‘flare’ states as detailed in the results section and Figure. There are no significant differences in subscales III and V among the ASD groups.

^b^Results of the ABC subscale and CSHQ total scores are expressed as average value ± standard deviation (SD).

^c^Results of the VABS was expressed as a number of subjects whose adaptive scale are <1st percentile rank (PR).

^d^ *Abbreviations used*; ASD, autism spectrum disorder; ASD-IS; ASD-inflammatory subtype; NFA, non-IgE mediated food allergy.

### Cytokine production by peripheral blood mononuclear cells versus peripheral blood monocytes

**Figure 2:**
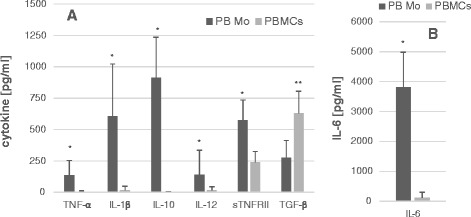
**Comparison of production of cytokines (TNF-α, IL-1β, IL-10, IL-12p40, sTNRII, and TGF-β) (Panel A) and IL-6 (Panel B) by purified peripheral blood monocytes (PB Mo) (0.25 × 10** ^**6**^ **cells/ml) and peripheral blood mononuclear cells (PBMCs) (10** ^**6**^ **cells/ml) from the same blood samples of randomly selected subjects from all the study groups (N = 21).** Each data point was shown as a mean cytokine level ± SD. The concentration of PB Mo and PBMC monocytes in culture were set to be approximately the same. *; *P* <0.001, **; *P* <0.005 (Wilcoxon rank sum test).

Initially, we tested whether purified PB Mo revealed cytokine production profiles similar to those we had seen before with the use of PBMCs. The concentration of PB Mo cells (0.25 × 10^6^ cells/ml) was adjusted to be approximately equivalent to the concentrations of PB Mo in PBMC cell cultures (10^6^ PBMCs/ml). PB Mo spontaneously produced significantly higher amounts of most cytokines measured, except for TGF-ß, than PBMCs (Figure 2).

### Cytokine production by peripheral blood monocytes without stimuli or in responses to stimuli

Since significant changes in ABC subscale scores between the ‘flare’ and ‘non-flare’ states were observed in ASD-IS subjects (Figure 1), we examined if changes in cytokine production also occurred in ‘flare’ versus ‘non-flare’ states in ASD-IS subjects.

#### Cytokine production in the absence of stimuli

**Figure 3:**
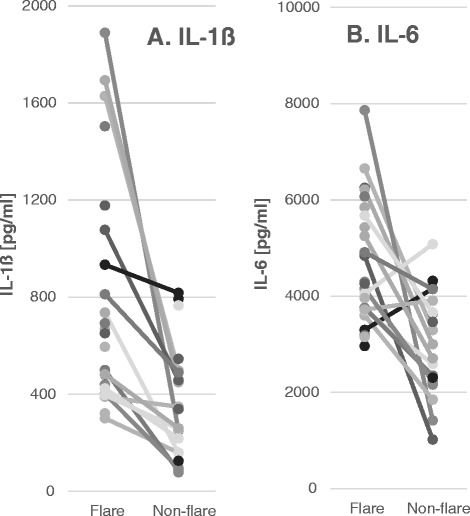
**Production of IL-1ß (Panel A) and IL-6 (panel B) by autism spectrum disorder-inflammatory subtype** **(ASD-IS) cells in ‘flare’ and non-flare’ states.** Levels of IL-1ß and IL-6 produced spontaneously by peripheral blood monocytes (PB Mo) were higher when in ‘flare’ than during ‘non-flare’ states in ASD-IS children (*P* < 0.01for IL-1ß and *P* <0.005 for IL-6).

Spontaneous production of IL-1ß and IL-6 by ‘flare’ ASD-IS cells was higher than ‘non-flare’ ASD-IS cells in matched pair analysis (Tables 4 and 5 and Figure 3). These levels were also higher than in most of the control cells (Tables 4 and 5). It should be noted that PANS cells revealed higher spontaneous production of IL-1β, but there were no difference in the spontaneous production of IL-1β or IL-10 between ‘flare’ versus ‘non-flare’ states (*P* >0.05). The same definition described for ASD-IS subjects, was used when assessing ‘flare’ versus ‘non-flare’ states in PANS subjects. In all the PANS subjects, ‘flare’ states were induced by microbial infection (mainly viral syndrome).

**Table 4:** **IL-1β production by purified peripheral blood monocytes in the study groups**

**Study group**	**Without stimuli [pg/ml]**	**LPS**	**Zymosan**	**CL097**
ASD-IS^d^				
- Flare	736.6 ± 242.4^a^	2141.2 ± 819.4^b^	2665.0 ± 569.5^b^	3062.0 ± 888.2
- Non-flare	424.8 ± 328.2	1596 ± 57.5	1990.4 ± 664.7	2925.8 ± 862.0
ASD/NFA	404.2 ± 242.4	582.7 ± 427.7^c^	373.7 ± 1620.7^c^	1056.8 ± 572.6^c^
ASD/non-NFA	471.9 ± 299.1	1592.8 ± 599.4	566.3 ± 1823.2	2729.2 ± 1025.4
Non-ASD/NFA	639.0 ± 424.0	1557.5 ± 566.6	1826 ± 678.2	2373.6 ± 1220.0
Normal control	361.2 ± 316.8	1580.4 ± 428.5	1709.9 ± 597.2	2668.7 ± 609.1
PANS				
- Flare	797.0 ± 598.8^a^	2310.9 ± 1057.3	2124.2 ± 590.4	3065.1 ± 683.8
- Non-Flare	731.9 ± 427.0^a^	1980.8 ± 845.6	1888.1 ± 630.2	2615.6 ± 791.6

^a^Spontaneous production of IL-1β is higher in ‘flare’ ASD-IS cells than in ‘non-flare’ ASD-IS cells in matched pair analysis as shown in Figure 3. IL-β levels produced spontaneously is also higher than those produced by normal, ASD/NFA, and ASD/non-NFA (*P* <0.02 for ‘flare’ ASD-IS cells and *P* <0.05 for PANS cells).

^b^IL-1ß levels produced by ‘flare’ ASD-IS cells in response to LPS and zymosan are higher than those by non-flare ASD-IS cells by matched pair analysis as shown in Figure 4. These levels are also higher than those produced by other control cells except for PANS cells.

^c^IL-1ß production by ASD/NFA cells in response to LPS, zymosan, and CL097 are lowest among the study groups (*P* <0.001).

^d^ *Abbreviations used*; ASD, autism spectrum disorders, ASD-IS; ASD-inflammatory subtype, IL, interleukin; LPS, lipopolysaccharide; NFA, non-IgE mediated food allergy; PANS, pediatric acute-onset neuropsychiatric syndrome.

**Table 5:** **IL-6 production by purified peripheral blood monocytes in the study groups**

**Study group**	**Without stimuli [pg/ml]**	**LPS**	**Zymosan**
ASD-IS			
- Flare	4541.7 ± 1332.8^a^	20662 ± 6181	936.6 ± 575.1^b^
- Non-Flare	3136.6 ± 1276.7	19420 ± 7051	1235.4 ± 1217
ASD/NFA	3682.7 ± 1613.3	17708 ± 10952	1352.4 ± 831.6
ASD/non-NFA	3827.3 ± 1467.4	18632 ± 10615	1281.7 ± 1068.5
Non-ASD/NFA	4029.8 ± 1246.1	20040 ± 6837	1334.0 ± 1031.4
Normal control	3102.8 ± 1225.4	17919 ± 10845	1716.1 ± 942.7
PANS			
- Flare	4005.0 ± 1512.2	22455 ± 9097	1123.9 ± 1487.5
- Non-Flare	3726.9 ± 1143.0	19663 ± 9718	871.6 ± 1013.0

^a^Spontaneous IL-6 production by ‘flare’ ASD-IS cells was higher than ‘non-flare’ cells in matched pair analysis, as shown in Figure 4 and also higher than those produced by ASD/NFA and normal control cells.

^b^IL-6 production by ‘flare’ ASD-IS cells in response to zymosan was lower than those produced by normal control cells (*P* <0.02) and also lower than the ‘non-flare’ ASD-IS cells in matched pair analysis (*P* <0.01).

^c^ *Abbreviations used*; ASD, autism spectrum disorder; ASD-IS, ASD-inflammatory subtype; IL, interleukin; LPS, lipopolysaccharide; NFA, non-IgE mediated food allergy; PANS, pediatric acute-onset neuropsychiatric syndrome.

#### Cytokine production in response to stimuli

**Figure 4:**
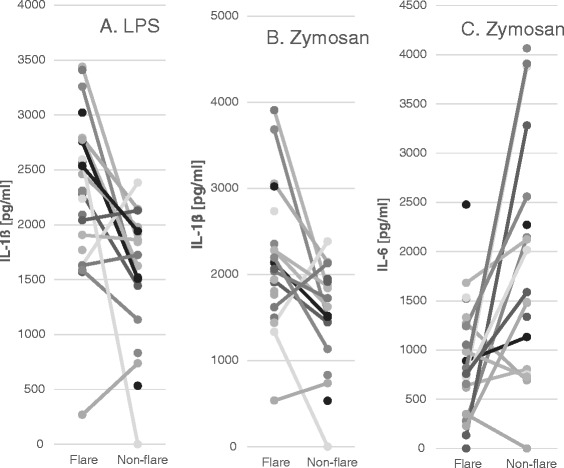
**IL-1ß produced in response to lipopolysaccharide (LPS) (Panel A) and zymosan (Panel B) and IL-6 in response to zymosan (Panel C) by ‘flare’ and ‘non-flare’ autism spectrum disorder-inflammatory subtype** **(ASD-IS) cells.** The values were expressed as IL-1ß or IL-10 produced with stimuli minus the values of IL-1ß or IL-10 produced spontaneously. IL-1ß production was higher by ‘flare’ ASD-IS cells than in ASD-IS ‘non-flare’ cells (*P* <0.005 and *P* <0.01, respectively). IL-6 production was lower in ‘flare’ ASD-IS cells than ‘non-flare’ cells (*P* <0.01) by Wilcoxin rank sum test.

Because of the high levels of spontaneous cytokine production by PB Mo, levels of most of the cytokines produced by PB Mo in response to stimuli were evaluated after subtracting the values of the cytokines produced spontaneously. The notable differences between the ‘flare’ versus ‘non-flare’ states of ASD-IS subjects were seen in the production of IL-1ß and IL-6, as summarized in Tables 4 and 5. Namely, IL-1ß production in response to LPS and zymosan was higher in the ‘flare’ ASD-IS cells than non-flare cells by matched-pair analysis (Figure 4) and also higher than cells from most control groups except for PANS cells. Although PANS cells tended to produce larger amounts of IL-1ß with stimuli, there were no significant differences in IL-1ß production in the ‘flare’ versus ‘non-flare’ states (Table 4). It should be noted that, among the study groups tested, the lowest production of IL-1ß was observed in the ASD/NFA subjects (Table 4). In contrast, IL-6 production in response to stimuli was lower in response to zymosan in ‘flare’ ASD-IS cells than in ‘non-flare’ ASD-IS cells (Figure 4 and Table 5), and this was also lower than that in normal control cells.

**Figure 5:**
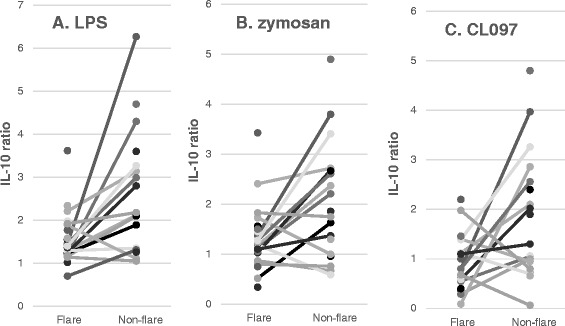
**Production of IL-10 in response to lipopoloysaccharide (LPS) (Panel A), zymosan (Panel B), and CL097 (Panel C) by autism spectrum disorder-inflammatory subtype (ASD-IS) peripheral blood (PB) monocytes.** The data are expressed as ratios of IL-10 produced with stimuli divided by IL-10 produced in the absence of stimuli. ASD-IS cells revealed lower values in the ‘flare’ state than in the ‘non-flare’ state (*P* <0.005 for LPS, *P* <0.05 for zymosan and CL097 by Wilcoxon rank sum test).

For the analysis of the IL-10 data, subtracting the spontaneous production of IL-10 could not be done. This is because IL-10 production with stimuli, especially with CL097 (TLR7/8 agonist), was lower than spontaneous IL-10 production in substantial numbers of study subjects. Thus, the results of IL-10 production were analyzed using the ratio of IL-10 produced with stimuli and IL-10 produced in the absence of stimuli. When the results were analyzed in this way, we found lower ratios of IL-10 in the ‘flare’ state of ASD-IS cells in response to LPS, zymosan, and CL097 than most control groups (Table 6). Matched-pair analysis also revealed significantly lower IL-1 ratio in the ‘flare’ ASD-IS cells than by ‘’non-flare’ ASD-IS cells (Figure 5). That is, ASD-IS ‘flare’ cells revealed significantly lower production of IL-10 as compared to all the control groups.

**Table 6:** **IL-10 production by purified peripheral blood monocytes in the study groups**

**The study group**	**Without stimuli [pg/ml]**	**LPS ratio** ^**a**^	**Zymosan**	**CL097**
ASD-IS^c^				
- Flare	848.6 ± 344.8	1.48 ± 0.88^b^	1.15 ± 0.43^b^	0.55 ± 0.63^2^
- Non-Flare	648.7 ± 382.1	2.64 ± 1.37	2.15 ± 1.09	1.23 ± 1.12
ASD/NFA	812.4 ± 365.5	1.96 ± 0.91	1.55 ± 0.75	0.89 ± 0.72
ASD/non-NFA	855.4 ± 364.7	1.93 ± 1.12	1.63 ± 1.03	0.93 ± 0.91
Non-ASD/NFA	948.5 ± 362.7	1.93 ± 1.38	1.66 ± 1.67	0.67 ± 0.51
Normal control	677.7 ± 290.0	2.40 ± 1.10	1.93 ± 1.06	0.76 ± 0.69
PANS				
- Flare	791.7 ± 449.4	1.87 ± 0.95	1.82 ± 1.27	1.38 ± 0.99
- Non-Flare	945.8 ± 455.4	1.93 ± 1.14	1.66 ± 0.84	1.58 ± 1.07

^a^IL-10 production in response to LPS, zymosan, and CL097 were expressed as a ratio of IL-10 produced in these stimulants/IL-10 produced in the absence of stimuli. It should be noted that IL-10 production tends to be lower in the presence of CL097 in most of the study groups.

^b^IL-10 production by ‘flare’ ASD-IS cells were lower than ‘non-flare’ ASD-IS cells with matched pair analysis as detailed in Figure IL-10 production by ‘flare’ ASD-IS cells in response to LPS and zymosan were also lower than ASD/NFA and normal control cells (*P* <0.01). IL-10 production by ‘flare’ ASD-IS cells in response to CL097 cells were also lower than those produced by PANS cells (*P* <0.01).

^c^ *Abbreviations used*; ASD, autism spectrum disorder; ASD-IS, ASD-inflammatory subtype; IL, interleukin; LPS, lipopolysaccharide; NFA, non-IgE mediated food allergy; PANS, pediatric acute-onset neuropsychiatric syndrome.

## Discussion

This study addressed cytokine production profiles by PB Mo in subgroups of ASD subjects categorized on the basis of clinical features and allergy/immunology s as detailed in the method section. To our knowledge, previous studies addressing PB Mo functions in ASD children did not subdivide ASD subjects and involved smaller numbers of ASD subjects than this study [26]. Also novel to this study, three non-ASD controls groups were included, that is, non-ASD/NFA, PANS, and typically developing normal controls without NFA or PANS. This experimental design permits us to dissect what findings are specific for the ASD-IS subjects. In addition, we analyzed the data depending on the ‘flare’ versus ‘non-flare’ states in the ASD-IS and PANS subjects. This is for testing our hypothesis that changes in cytokine production profiles in PB Mo can be associated with fluctuating behavioral symptoms following immune insults in the ASD-IS and PANS children.

In this study, parental impression of behavioral changes in ASD-IS children following immune insults were verified first, using the ABC and CSHQ. Behavioral symptoms, per parental reports, would typically ‘flare’ for a prolonged period following immune insults in ASD-IS children, despite resolution of acute physical symptoms such as fever or other signs of acute inflammation. When these results were compared to baseline data obtained during ‘non-flare’ states, we found significantly higher scores in subdomains I (irritability), II (lethargy), and IV (hyperactivity) in ABC scores (Table 3 and Figure 1) during ‘flares’ in ASD-IS children. These findings support parental impression of worsening behavioral symptoms in the ‘flare’ state following immune insults. In the ASD-IS children, ABC scores were assessed after resolution of acute symptoms of immune insults such as upper respiratory infection. Therefore, it is unlikely that behavioral changes are solely attributed to the pain and/or discomfort associated with acute illnesses.

Mounting evidence indicates that in other neuropsychiatric conditions such as schizophrenia, inflammatory cytokines such as IL-1β and IL-6 have a role in onset and progress of neuropsychiatric symptoms [27-31]. We have reported previously that PBMCs obtained from ASD-IS children produced reduced amounts of pro-inflammatory cytokines (IL-1β and IL-6) and IL-10, a counter-regulatory cytokine [1]. In these studies, most samples were obtained when ASD-IS children were in the ‘non-flare’ states. Thus, the altered PB Mo functions following a ‘flare’ may have not been captured in our previous study. Alternatively, other lineage cells, other than PB Mo, may have suppressed the functions of PB Mo. In order to address these functions of PB Mo more precisely, this study employed purified PB Mo from ASD-IS children in the ‘flare’ versus ‘non-flare’ states.

First, we assessed the differences in cytokine production profiles between PBMCs and PB Mo. We measured cytokine production by PBMCs and PB Mo prepared from the same blood samples in 21 subjects randomly selected from all the study groups. As shown in Figure 2, PB Mo spontaneously produced higher amounts of most cytokines measured except for TGF-β. The concentration of PB Mo in the monocyte cultures was then adjusted to be roughly equivalent to that of the monocyte concentration in PBMC cultures. Thus, it is very unlikely that changes in cell concentrations are responsible for the differences in spontaneous production of cytokines by PBMCs and PB Mo. Instead, it is more likely that cytokine production by PB Mo is suppressed by other lineage cells present in PBMCs. For example, counter-regulatory cytokines such as TGF-β and IL-10 produced by inducible regulatory T (Treg) cells are known to suppress functions of effecter cells including Mo [32]. The fact that production of TGF-β, one of the major regulatory cytokines in the periphery, was higher than that produced by PB Mo indicates a possibility that TGF-β produced by other lineage cells suppress spontaneous cytokine production by PB Mo. Based on these findings, we reasoned that the use of purified PB Mo will likely yield more information regarding changes in monocyte function in ASD-IS children.

Spontaneous production of cytokines by PB Mo was then analyzed in all the study groups. The data from ASD-IS and PANS children were analyzed in the ‘flare’ versus ‘non-flare’ states. PANS children are characterized by acute onset neuropsychiatric changes following immune insults (typically microbial infection) [18]. In our experience, ASD-IS children are often diagnosed with PANS on the basis of their fluctuating behavioral symptoms following immune insults. In the PANS children recruited to this study, triggers of acute onset of neuropsychiatric symptoms varied from viral infection to bacterial infection, and none of them qualified for diagnosis of PANDAS (pediatric autoimmune neuropsychiatric diseases associated with streptococci) [13].

Our results of monocyte cytokine profiles revealed higher spontaneous production of inflammatory cytokines (IL-6, and IL-1β) in the ‘flare’ state than in the ‘non-flare’ states in ASD-IS children (Tables 4 and 5). In PANS children, such changes were not observed. When purified PB Mo were further stimulated by various stimuli of innate immunity, we found that despite increased spontaneous production of IL-1β, ‘flare’ ASD-IS cells are still capable of producing higher amounts of IL-1ß in response to LPS and zymosan than ‘non-flare’ ASD-IS cells. IL-1ß production by ‘flare’ ASD-IS cells are also higher than other ASD/non-ASD control cells, except for PANS cells. In contrast, IL-6 production by ‘flare’ ASD-IS cells in response to stimuli was equivalent or even lower than that of ‘non-flare’ ASD-IS cells (Table 5 and Figure 4-C). It should also be emphasized that such changes were not observed in PANS children in ‘flare’ versus ‘non-flare’ states, although they tended to exhibit increased production of IL-1ß by PB Mo.

When in an inflammatory state or when subjects are recovering from inflammation, proinflammatory immune responses are generally suppressed by intrinsic suppressive mechanisms of cells or by other lineages cells with suppressive functions. This will help self-limit the inflammatory processes. However, our results indicates that ASD-IS PB Mo are still capable of producing significant amounts of IL-1ß in response to stimuli even in the ‘flare state, despite spontaneous production of high amounts of IL-1ß. Since our assay used the purified PB Mo, our results indicate that ASD-IS monocytes have impaired suppressive mechanisms for IL-1ß production following activation. IL-6 production did not increase in response to stimuli in ‘flare’ ASD-IS cells. Production of functional IL-1ß requires additional enzymatic steps as opposed to other cytokines [33]. Therefore, it is possible that ASD-IS children have impaired regulation in the specific steps of IL-1ß production.

Dysregulated production of IL-1β and IL-6 has been known to lead to chronic inflammatory conditions of the CNS, joints, and GI tracts. For example, in cryopyrin associated periodic fever syndrome (CAPS), autosomal dominant mutation of cryopyrin leads to excessive production of IL-1β, leading to chronic systemic inflammatory condition affecting the CNS, joints, and other organs [34]. In juvenile idiopathic arthritis (JIA), excessive production of IL-6 and IL-1β (for systemic JIA) has been implicated in their pathogenesis [35]. In these conditions, blockers of these inflammatory cytokines have been effective for treating these conditions [35,36]. Interestingly, in a rodent maternal immune activation (MIA) model for autism, a single injection of IL-6 to pregnant mice at gestational age of 12.5 days was reported to result in deficits in certain behaviors in the adult offspring [37]. Likewise, increased serum levels of IL-1β in offspring have also been reported in MIA models [38]. In patients with primary autoinflammatory conditions causing excessive action of IL-1ß, CNS symptoms that partly resemble ASD behavioral symptoms are well controlled by exogenous IL-1 blockers [34]. These reports along with our results indicate a possibility that changes in IL-1ß responses may be associated with changes in behavioral symptoms observed in ASD-IS children following immune insults. These findings further support the need for mechanistic studies to assess the feasibility for IL-1ß and/or IL-6 blockage strategy as a strategy for treating ASD-IS children.

In chronic inflammation, IL-10, a counterregulatory cytokine, is generally upregulated as part of the immunoregulatory actions [39]. However, in the ASD-IS children, spontaneous IL-10 production did not differ between the ‘flare’ and ‘non-flare’ states. In addition, we found a significant decrease in IL-10 production by ‘flare’ ASD-IS cells in response to stimuli of innate immunity as compared to ‘non-flare’ ASD-IS cells (Figure 5). This was not found in PANS children. Production of IL-10, one of the key regulators of inflammatory responses, by PB Mo is an important intrinsic regulatory mechanism for controlling excessive inflammation [39]. In the state of microbial infection, IL-10 production is often downregulated to mount an immune defense effectively. However, excessive, prolonged downregulation of IL-10 could cause dysregulated, prolonged inflammation. Thus, our results indicate a possibility that monocytes from ASD-IS children also have intrinsic defects in regulatory mechanisms of IL-10 production.

Lastly, it should be noted that the cytokine production profiles in PB Mo differed between ASD-IS and PANS subjects in this study. This finding indicates that although fluctuating behavioral symptoms are somewhat overlapping in ASD-IS and PANS subjects, underlying mechanisms may not be identical in these two groups.

## Conclusions

In summary, studies of purified PB Mo from ASD-IS children indicate that they have dysregulated innate immune responses. Specifically, these relate to the production of IL-1ß and IL-10, revealing significant changes in ‘flare’ versus ‘non-flare’ states. These responses, for the most part, were not observed in ASD/non-ASD controls. These results indicate that further analysis of the regulatory mechanisms of PB Mo in ASD-IS subjects may lead to the identification of biomarkers and even treatment options for ASD-IS subjects who typically do not respond well to the first-line therapeutic measures.

## Abbreviations

Ab: antibody
ABC: Aberrant Behavior Checklist
AC: allergic conjunctivitis
ADI-R: Autism Diagnostic Inventory-Revisited
ADOS: Autism Diagnostic Observation Scale
AR: allergic rhinitis
ASD: autism spectrum disorder
ASD-IS: autism spectrum disorder-inflammatory subtype
CAM: complementary alternative medicine
CSHQ: Children’s Sleep Habit Questionnaire
DC: dendritic cells
EoE: eosinophilic esophagitis
FA: food allergy
FPIES: food protein induced enterocolitis syndrome
GI: gastrointestinal
GIA: general intellectual ability
IL: interleukin
LPS: lipopolysaccharide
MIA: maternal immune activation
miRNA: micro RNA
Mo: monocytes
NFA: non-IgE mediated food allergy
PANS: pediatric acute-onset neuropsychiatric syndrome
PB: peripheral blood
PBMCs: peripheral blood mononuclear cells
SPAD: specific polysaccharide antibody deficiency
sTNFRII: soluble tumor necrosis factor receptor II
TGF-ß: transforming growth factor-ß
TLR: toll-like receptor
Treg cells: regulatory T cells
VABS: Vineland Adaptive Behavioral scale
WJIII: Woodcock-Johnson III
